# Impact of Natalizumab on Ambulatory Improvement in Secondary Progressive and Disabled Relapsing-Remitting Multiple Sclerosis

**DOI:** 10.1371/journal.pone.0053297

**Published:** 2013-01-04

**Authors:** Diego Cadavid, Stephanie Jurgensen, Sophia Lee

**Affiliations:** 1 MS Clinical Development Group, Biogen Idec, Cambridge, Massachusetts, United States of America; 2 MS Global Medical Affairs, Genzyme, a Sanofi Company, Cambridge, Massachusetts, United States of America; 3 Biometrics, Biogen Idec, Cambridge, Massachusetts, United States of America; Charite Universitätsmedizin, Germany

## Abstract

**Background:**

There is an unmet need for disease-modifying therapies to improve ambulatory function in disabled subjects with multiple sclerosis.

**Objectives:**

Assess the effects of natalizumab on ambulatory function in disabled subjects with relapsing-remitting multiple sclerosis (RRMS) or secondary progressive multiple sclerosis (SPMS).

**Methods:**

We retrospectively reviewed ambulatory function as measured by timed 25-foot walk (T25FW) in clinical trial subjects with an Expanded Disability Status Scale score ≥3.5, including RRMS subjects from the phase 3 AFFIRM and SENTINEL trials, relapsing SPMS subjects from the phase 2 MS231 study, and nonrelapsing SPMS subjects from the phase 1b DELIVER study. For comparison, SPMS subjects from the intramuscular interferon beta-1a (IM IFNβ-1a) IMPACT study were also analyzed. Improvement in ambulation was measured using T25FW responder status; response was defined as faster walking times over shorter (6–9-month) or longer (24–30-month) treatment periods relative to subjects’ best predose walking times.

**Results:**

There were two to four times more T25FW responders among disabled MS subjects in the natalizumab arms than in the placebo or IM IFNβ-1a arms. Responders walked 25 feet an average of 24%–45% faster than nonresponders.

**Conclusion:**

Natalizumab improves ambulatory function in disabled RRMS subjects and may have efficacy in disabled SPMS subjects. Confirmation of the latter finding in a prospective SPMS study is warranted.

## Introduction

Decreased ambulatory function is an important manifestation of impairment in multiple sclerosis (MS) and contributes to loss of quality of life and independence, as well as restriction of activities of daily living [1], [2]. Loss of ambulatory function in MS can occur as a result of incomplete recovery from clinical exacerbations, as in relapsing-remitting MS (RRMS), or as a result of steady accumulation of disability, as in secondary progressive MS (SPMS) and primary progressive MS (PPMS). Disease-modifying therapies that prevent loss of or improve ambulatory capacity in disabled MS subjects are needed to increase independence in daily living activities and enhance quality of life. Recently, prolonged-release (PR) fampridine (Fampyra®, Biogen Idec, Weston, Massachusetts, USA; known in the United States as dalfampridine extended release, Ampyra®, Acorda Therapeutics, Ardsley, NY, USA) was approved in the European Union and the United States as the first symptomatic treatment to improve walking in patients with MS. In the phase 3 PR-fampridine studies, the primary endpoint was the proportion of responders, defined as patients with faster walking speed on at least three of four on-treatment assessments compared with the fastest of five off-treatment assessments. Approximately one-third (37%) of treated patients were responders; in these patients, average walking speed on therapy was about 25% faster than baseline [3], [4].

In the pivotal AFFIRM and SENTINEL clinical trials in RRMS, natalizumab (Tysabri®, Biogen Idec, Weston, Massachusetts, USA, and Elan Pharmaceuticals, South San Francisco, California, USA) reduced the annualized relapse rate by 68% (*P<*0.001) as monotherapy [5] and by 55% (*P<*0.001) when used in combination with weekly intramuscular (IM) injections of interferon beta-1a (IFNβ-1a) [6]. Progression of disability was also reduced by 42% in AFFIRM [5] and by 24% in SENTINEL [5], [6]. Recent post hoc analyses demonstrated improvement in disability in RRMS subjects in AFFIRM [7]. Natalizumab is in rare cases associated with progressive multifocal leukoencephalopathy, which can lead to death and severe disability [8].

Here we investigated the possibility that natalizumab may improve ambulatory function in disabled RRMS and SPMS subjects. The traditional measurements of ambulatory function in MS clinical trials are the 500-meter walk component of the Expanded Disability Status Scale (EDSS) [9] and the timed 25-foot walk (T25FW) component of the Multiple Sclerosis Functional Composite (MSFC) [10]. Analyses from the SPMS IMPACT and PPMS OLYMPUS trials found that the T25FW is more responsive to change in ambulatory function in disabled MS subjects than the EDSS [11], [12]. Therefore, for the present analyses, we used the T25FW to investigate natalizumab’s effects on ambulatory capacity in disabled RRMS and SPMS subjects.

## Subjects and Methods

All subjects included in this retrospective analysis had participated in previous clinical studies (AFFIRM,^5^ SENTINEL,^6^ IMPACT,^11^ OLYMPUS,^12^ and DELIVER). The protocols for the original studies were approved by independent ethics committees for all participating study centers. (Appendix S1 lists the independent ethics committees/institutional review boards for centers participating in the DELIVER study, which has not been published.) The studies were performed in accordance with the Declaration of Helsinki and Good Clinical Practice guidelines, and all patients provided written informed consent.

### Disabled RRMS Subjects

We evaluated RRMS subjects from AFFIRM (ClinicalTrials.gov NCT00027300) [5] and SENTINEL (ClinicalTrials.gov NCT00030966) [6] with baseline EDSS score ≥3.5, T25FW >5 seconds, and at least one pretreatment and one posttreatment T25FW assessment. AFFIRM was a double-blind trial of RRMS subjects randomized 2∶1 to receive 300 mg of natalizumab or placebo by intravenous (IV) infusion every 4 weeks for up to 30 months [5]. SENTINEL was a 30-month double-blind trial of RRMS subjects randomized 1∶1 to receive IV infusions of placebo every 4 weeks or 300 mg of natalizumab as an add-on therapy to the 30 µg IM IFNβ-1a subjects had been taking weekly for at least 1 year. The present analysis included 50 subjects randomized to placebo and 97 subjects randomized to natalizumab in AFFIRM and 109 subjects randomized to IFNβ-1a plus placebo and 102 subjects randomized to IFNβ-1a plus natalizumab in SENTINEL.

### SPMS Subjects

We evaluated subjects with at least one pretreatment and one posttreatment T25FW assessment from two natalizumab trials and one IFNβ-1a trial. The first set of subjects, from the phase 2 MS231 trial [13], was randomized 1∶1:1 to receive placebo or 3 or 6 mg/kg of natalizumab IV monthly for 6 months, with 6 additional months of follow-up. For the current post hoc analyses, all relapsing SPMS subjects randomized to either 3 mg/kg (n = 21) or 6 mg/kg (n = 20) of natalizumab were analyzed together (n = 41) and compared with the 25 subjects with relapsing SPMS randomized to placebo. The second set of subjects, from the open-label phase 1b DELIVER trial (ClinicalTrials.gov NCT00559702) (101MS102), included 52 subjects with nonrelapsing SPMS randomized 2∶2:2∶1 to receive natalizumab 300 mg IV (n = 16), subcutaneous (SC, n = 14), or IM (n = 15) for 8 months or standard of care (symptomatic treatment, n = 7) over the same span. For the present analyses, all subjects from DELIVER randomized to natalizumab were pooled together (n = 45). The standard of care group was excluded because of the small number of subjects with posttreatment T25FW (n = 5) and the marked difference between this group’s mean baseline T25FW (24.1 seconds) and that of the natalizumab groups (14.4 seconds). The third set of subjects were randomized 1∶1 to receive weekly IM injections of 60 µg of IFNβ-1a (n = 210) or placebo (n = 213) in the IMPACT trial [14]. IMPACT included SPMS subjects regardless of relapse rate.

### Measurement of Improvement in Ambulatory Function

T25FW was performed according to the MSFC protocol in all five trials [10], [15], though with variable frequency and duration: at baseline and every 12 weeks for 1 year (MS231); at baseline and at weeks 8, 20, and 32 (DELIVER); or at baseline and every 12 weeks for 2 years (IMPACT) or 30 months (AFFIRM and SENTINEL). Improvement in ambulatory capacity was measured using a responder analysis based on consistency of improvement upon regular repeated testing, analogous to what was done in the PR-fampridine clinical trials [3], [4], albeit at longer intervals. T25FW responders were defined as subjects who walked faster in the majority of postbaseline visits than in their best baseline T25FW. We used the best baseline T25FW rather than the average of two baseline walks specified in the standard MSFC to be more stringent in our definition of responders. We preferred consistent improvement over the course of several assessments over absolute improvement at single time points because of the well-known fluctuation in ambulatory performance of disabled MS subjects. The exact responder criterion for each trial was dependent upon treatment duration and the number of assessments: in the shorter-term phase 1/2 trials, responders were defined as having improvement in at least 2 of 3 visits over 6–8 months, whereas in the longer-term phase 3 trials, responders were defined as having improvement in at least 6 of 8 quarterly visits over 24 months in IMPACT or at least 7 of 10 quarterly visits over 30 months in AFFIRM and SENTINEL. For these longer-term trials, we also determined whether improvement was already apparent during shorter-term treatment by defining T25FW responders as those subjects exhibiting improvement in their 2 visits over the first 6 months. To determine the degree of improvement in responders and nonresponders, we assessed the percentage improvement in T25FW from baseline to the 8–9-month visits for shorter-term trials and from baseline to the 24–30-month visits for longer-term trials. We also did sensitivity analyses by varying the definition of T25FW responders over 30 months to require different levels of consistency in improvement (e.g., improvement in 6 of 10, 7 of 10, or 8 of 10 visits) and by evaluating patients with baseline T25FW >7 seconds in addition to the >5-second T25FW threshold used for all other analyses.

### Statistical Analysis

All *P* values assessing the difference between treatment groups were calculated using a two-sided Fisher exact test.

## Results

### Baseline Characteristics

A total of 906 subjects were included in these analyses: 358 disabled RRMS subjects (from two trials) and 548 SPMS subjects (from three trials). Baseline characteristics are summarized in Table 1. The majority of subjects in both groups were female, but the RRMS subjects were younger than the SPMS subjects. Average EDSS scores were slightly lower in RRMS subjects than in SPMS subjects, and only SPMS subjects had EDSS scores >6.0. Preceding clinical relapsing activity was highest in RRMS and relapsing SPMS subjects. The number of enhancing brain lesions was lowest in nonrelapsing SPMS subjects from DELIVER and RRMS subjects from SENTINEL. Baseline T25FW averaged 14–17 seconds in SPMS subjects and approximately 8 seconds in RRMS subjects. For comparison, nondisabled MS subjects and healthy persons typically walk 25 feet in less than 5 seconds [16].

**Table 1:** Baseline subject characteristics.

	MS231	DELIVER	IMPACT	AFFIRM	SENTINEL
Clinical phase	2b	1b	3	3	3
Type of MS	Relapsing SPMS	Nonrelapsing SPMS	All forms of SPMS	Disabled RRMS, treatment naive	Disabled RRMS on IM IFNβ-1a >12 months
Treatment arms	Placebo or natalizumab	Natalizumab	Placebo or IM IFNβ-1a	Placebo or natalizumab	Add-on placebo or natalizumab
Number of subjects	66	45	423	147	211
Age, years, mean (SD)	47.3 (8.33)	53.8 (6.86)	47.7 (7.9)	39.5 (7.20)	41.7 (6.89)
Percentage female	68	71	64	69	77
EDSS score, mean (SD)	5.67 (0.99)	5.66 (1.20)	5.20 (1.10)	4.11 (0.61)	4.09 (0.56)
Percentage of subjects withEDSS score >6.0	29	42	24	0	0
Number of relapses in prior36 months, mean (SD)^a^	2.8 (1.29)	0.7 (1.04)	1.4 (2.2)	3.1 (1.80)	3.1 (1.66)
Time since last relapse, months,mean (SD)	6.7 (3.33)	69.7 (54.90)	49.8 (60.7)	6.6 (2.94)	6.0 (2.96)
Baseline number of Gd+ lesions,mean (SD)	1.52 (2.64)	0.20 (0.59)	1.4 (4.68)	2.20 (5.27)	0.80 (1.51)
Baseline T25FW, seconds,mean (SD) [median (range)]	17.0 (23.6) [10.55 (4.0–122.8)]	14.4 (10.6) [11.15 (4.6–61.0)]	14.0 (15.8)[9.1 (3.1–140.8)]	7.6 (3.3) [6.75 (5.1–32.9)]	8.0 (5.1) [6.55 (5.1–58.8)]

a Number of relapses in prior 24 months for MS231.

MS, multiple sclerosis; SPMS, secondary progressive MS; RRMS, relapsing-remitting MS; IM, intramuscular; IFNβ-1a, interferon beta-1a; SD, standard deviation; EDSS, Expanded Disability Status Scale; Gd+, gadolinium enhancing; T25FW, timed 25-foot walk.

### T25FW Improvement During Shorter-term Natalizumab Treatment

We evaluated the percentage of T25FW responders following shorter-term treatment (6–9 months). The proportion of shorter-term responders was highest (*P* = not significant) in the natalizumab arms of the SPMS studies (Table 2): 24% in the relapsing SPMS study MS231 and 22% in the nonrelapsing SPMS DELIVER study. By comparison, only 12% of subjects in the placebo group of study MS231 were responders over the shorter term. Low percentages of shorter-term T25FW responders were also observed in the placebo and IFNβ-1a arms of the IMPACT SPMS study (8% and 11%, respectively). The proportion of shorter-term T25FW responders with RRMS was 16% in the natalizumab monotherapy arm versus 4% in the placebo arm (*P = *0.03) in AFFIRM, and 11% in the natalizumab plus IFNβ-1a arm versus 9% in the placebo plus IFNβ-1a arm in SENTINEL (*P = *not significant).

**Table 2:** Percentage of T25FW responders over shorter-term (6–8 months) and longer-term (24–30 months) follow-up in disabled RRMS^a^ and SPMS subjects during treatment with natalizumab compared with placebo or IM IFNβ-1a.

Study	Natalizumab	Placebo	IM IFNβ-1a	*P* value
**Shorter-term treatment**				
MS231	24% (10/41)	12% (3/25)		0.34
DELIVER	22% (10/45)	Not done		
IMPACT		8% (16/212)	11% (23/208)	0.24
AFFIRM	16% (16/97)	4% (2/50)		0.03
SENTINEL^b^	11% (11/102)	9% (10/109)		0.82
**Longer-term treatment**				
AFFIRM	22% (21/97)	10% (5/50)		0.11
SENTINEL^b^	19% (19/102)	7% (8/109)		0.02
IMPACT		7% (15/213)	8% (16/210)	0.85

a Disabled RRMS subjects defined as those with EDSS ≥3.5 and T25FW >5 seconds at baseline.

b In SENTINEL, subjects on IM IFNβ-1a for at least 12 months were randomized to adding natalizumab or placebo.

T25FW, timed 25-foot walk; RRMS, relapsing-remitting multiple sclerosis; SPMS, secondary progressive multiple sclerosis; IM, intramuscular; IFNβ-1a, interferon beta-1a; EDSS, Expanded Disability Status Scale.

### T25FW Improvement with Longer-term Natalizumab Treatment

The frequency of T25FW improvement during longer-term treatment with natalizumab was analyzed in the disabled RRMS subjects from AFFIRM and SENTINEL; since there are no longer-term natalizumab data in SPMS, the IFNβ-1a and placebo arms of the IMPACT SPMS study provided comparative data. The proportion of 30-month T25FW responders was 2 to 2.5 times higher in the natalizumab monotherapy and combination therapy with IFNβ-1a arms of AFFIRM and SENTINEL (19%–22%) than in the placebo and IFNβ-1a monotherapy arms of AFFIRM, SENTINEL, and IMPACT (7%–10%). The difference was significant only in the SENTINEL trial (Table 2).

### Differences in Ambulatory Function between T25FW Responders and Nonresponders

We calculated absolute and percentage changes from baseline over the shorter term (6–8 months) for RRMS and SPMS subjects classified as T25FW shorter-term responders versus nonresponders in all five trials. For this analysis, treatment groups were combined, and the results confirmed that T25FW responders did better over time than T25FW nonresponders (Table 3). The overall difference in mean (median) percentage change from baseline between T25FW responders and nonresponders was 24%–35% (20%–24%) in RRMS subjects compared with 35%–36% (17%–25%) in SPMS subjects. The overall difference between responders and nonresponders in change from baseline in T25FW over the shorter term was larger among SPMS subjects (5.0–8.9 seconds) than among RRMS subjects (1.7–4.8 seconds) (Table 3).

**Table 3:** Absolute and percentage change from baseline in T25FW between responders and nonresponders independent of treatment group over shorter-term (6–8 months) and longer-term (24–30 months) follow-up in disabled subjects with RRMS and SPMS.

Study	Time (months)	T25FW responder	n	Absolute change, seconds, mean (SD)^a^	Absolute change, seconds, median^a^	Percentage change, mean	Percentage change, median
**Shorter-term treatment**							
IMPACT	6	No	357	2.9 (11.75)	0.5	20	7
	6	Yes	39	−2.3 (3.06)	−1.3	−15	−13
DELIVER	8	No	36	4.8 (11.65)	0.6	26	6
	8	Yes	10	−0.2 (4.51)	−1.7	−10	−11
MS231	6	No	47	4.5 (12.00)	1.4	23	15
	6	Yes	13	−4.4 (12.02)	−1.1	−12	−10
AFFIRM	6	No	125	0.7 (2.92)	0.3	10	5
	6	Yes	18	−1.0 (0.78)	−1.0	−14	−15
SENTINEL	6	No	184	0.8 (2.54)	0.2	10	3
	6	Yes	21	−4.7 (9.15)	−1.6	−25	−21
**Longer-term treatment**							
IMPACT	24	No	321	7.0 (17.21)	1.5	54	20
	24	Yes	36	2.4 (8.41)	−0.1	20	−1
AFFIRM	30	No	108	1.2 (4.41)	0.5	18	7
	30	Yes	17	−0.8 (1.16)	−0.9	−11	−12
SENTINEL	30	No	144	1.9 (5.25)	0.9	26	13
	30	Yes	18	−4.7 (12.03)	−1.3	−18	−17

a In seconds.

T25FW, timed 25-foot walk; RRMS, relapsing-remitting multiple sclerosis; SPMS, secondary progressive MS; SD, standard deviation.

When data were available, we also explored whether the differences observed between responders and nonresponders over shorter-term follow-up (6–9 months) were evident in longer-term (24–30-month) follow-up. Shorter-term T25FW responders from AFFIRM took an average of 11% less time to walk 25 feet at 30 months than at baseline, while shorter-term nonresponders took 18% more time, an absolute difference of 29%. Similarly, shorter-term responders from SENTINEL took 18% less time to walk 25 feet at 30 months than at baseline, while nonresponders took 26% more time, an absolute difference of 44%. In IMPACT, shorter-term T25FW responders took an average of 20% more time at 24 months while nonresponders took 54% more time, an absolute difference of 34%. The difference between shorter-term responders and nonresponders in mean change from baseline during longer-term follow-up was 4.6 seconds among SPMS subjects and 2.0–6.6 seconds among RRMS subjects (Table 3).

### Sensitivity Analysis of T25FW Responder Status

**Figure 1:**
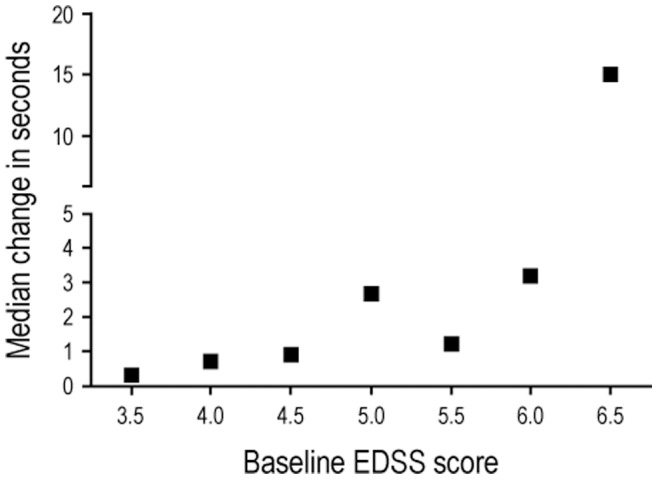
Median change in T25FW over 2 years by baseline EDSS score in patients with SPMS. Patients randomized to the placebo arm of the IMPACT study. T25FW, timed 25-foot walk; EDSS, Expanded Disability Status Scale; SPMS, secondary progressive multiple sclerosis.

We studied whether the effects of natalizumab on T25FW responder status in disabled RRMS subjects were consistent across different levels of ambulatory impairment at baseline and consistency of improvement over 2 years. Irrespective of whether T25FW responder status was defined as requiring consistent improvement over 6, 7, or 8 of 10 quarterly visits over 30 months, the percentage of T25FW responders remained higher with natalizumab than with placebo (Table 4). The treatment effects of natalizumab on T25FW responder status were stronger for subjects who were more impaired at baseline (those meeting the criterion of T25FW >7 seconds rather than simply T25FW >5 seconds). This is consistent with the finding that the T25FW is more responsive to change in subjects who are more disabled at baseline (Figure 1).

**Table 4:** Percentage of disabled RRMS subjects (EDSS score ≥3.5) with improvement in T25FW from best baseline walk over 30 months following randomization to natalizumab or placebo in the AFFIRM study: sensitivity analyses by consistency of improvement.

	Natalizumab	Placebo	*P* value
**Any improvement in ≥6 of 10 quarterly visits**			
Baseline T25FW >7 seconds	40% (17/43)	5% (1/21)	0.003
**Any improvement in ≥7 of 10 quarterly visits**			
Baseline T25FW >5 seconds	22% (21/97)	10% (5/50)	0.110
Baseline T25FW >7 seconds	33% (14/43)	5% (1/21)	0.014
**Any improvement in ≥8 of 10 quarterly visits**			
Baseline T25FW >5 seconds	15% (15/97)	8% (4/50)	0.300
Baseline T25FW >7 seconds	23% (10/43)	0% (0/21)	0.023

*P* values assessing the difference between treatment groups based on two-sided Fisher exact test.

RRMS, relapsing-remitting multiple sclerosis; EDSS, Expanded Disability Status Scale; T25FW, timed 25-foot walk.

## Discussion

A major consequence of disease activity in MS is loss of ambulatory capacity, which, during the early stages, may be more evident over longer distances but which, as disability progresses, becomes pronounced over shorter distances as well [17]–[20]. The etiology of ambulatory impairment may be related to spasticity, incoordination, sensory loss, fatigue, pain, and cognitive dysfunction [2], in addition to the direct pathological effects of MS on motor function. Impaired mobility significantly impairs activities of daily living and quality of life and often results in the loss of independence [1], [2]. There is a high unmet need for therapies that improve ambulatory capacity in MS.

We performed a post hoc analysis on T25FW data from cohorts of disabled MS patients from five different clinical trials to investigate whether natalizumab treatment may lead to ambulatory improvement. Data from shorter-term treatment durations (≈6–8 months) were available from all five trials; data from longer-term treatment durations (≈2 years) were available from three trials. The sample size from the SPMS trials was small. The main findings were as follows: (1) Improvement in ambulatory function as measured by the T25FW responder analysis was observed more frequently in disabled RRMS and SPMS subjects treated with natalizumab (alone or in combination with IFNβ-1a) than in subjects treated with IM IFNβ-1a monotherapy or placebo, although the differences reached significance only in the disabled RRMS groups. (2) The frequency of T25FW responder status during shorter-term treatment with natalizumab was similar in relapsing and nonrelapsing SPMS subjects. (3) T25FW responders took an average of 24%–44% less time to walk 25 feet than nonresponders. (4) Natalizumab consistently improved T25FW responder status in disabled RRMS subjects, with the strongest treatment effect seen in the most ambulatory-impaired subjects.

Consistent improvement in T25FW was observed with similar frequency in the two shorter-term SPMS trials, although one was enriched for recent relapses (MS231) while the other was enriched for the absence of relapses (DELIVER). The short duration and sample size of these two trials and the lack of a placebo arm in DELIVER limit interpretation of these findings. In fact, the differences between the SPMS groups were not statistically significant. In the longer-term data from disabled RRMS subjects, the proportion of responders was greater in the natalizumab arms than in the nonnatalizumab arms (Tables 2 and 4). However, a treatment effect was apparent over a shorter-term follow-up only in AFFIRM. This difference may be due to the higher frequency of relapsing inflammatory activity in AFFIRM; the baseline average gadolinium-enhancing lesion count was 2.2 in AFFIRM versus 0.8 in SENTINEL (Table 1). Interestingly, in the longer-term data, the difference was significant only for SENTINEL (Table 2).

There are many difficulties associated with investigating efficacy in SPMS patients in clinical trials. For this exploratory analysis that included shorter-term studies, we measured improvement using consistent improvement in T25FW. In a recent analysis, we showed that consistent worsening of T25FW was the most responsive clinical measure to disease progression in disabled MS patients in two large clinical trials [12]. The T25FW responder definitions did not include a minimum fixed threshold for improvement but rather accepted improvement if it was consistently observed from the best baseline measure in the majority of postbaseline assessments regardless of the magnitude of improvement. We believe that consistent improvement over periodic longitudinal assessments is a better approach than comparison of two time points because of day-to-day fluctuations in ambulation in disabled subjects with MS. Consistency of T25FW improvement was recently used in two placebo-controlled clinical trials to show treatment effects with the symptomatic therapy PR-fampridine in MS subjects with baseline T25FW ≥8 seconds [3], [4].

We found that the difference between responders and nonresponders in T25FW percentage change from baseline ranged from 24% to 44% across the five trials (Table 3). Worsening in T25FW was greater in SPMS subjects than in disabled RRMS subjects over the shorter-term follow-up (Table 3). This may be related to the greater responsiveness of T25FW to change over time in more disabled patients, which was reported in the initial MSFC publication [10] and confirmed in our analysis of the IMPACT placebo data in the current study (Figure 1). During the 9- to 14-week efficacy evaluation period in the PR-fampridine studies, responding subjects exhibited an average improvement of ≈25% from baseline in T25FW walking speed. About a third (33%) of the PR-fampridine–treated patients had improvements in walking speed of at least 20% [3], [4]. It has been proposed that the minimal important change in T25FW in MS is approximately 20% [21]–[23], which is less than the absolute difference observed between T25FW responders and nonresponders in our analyses.

Only about 15%–25% of natalizumab-treated patients were T25FW responders (Tables 2 and 3). This clinically important yet limited percentage of disabled MS subjects who may achieve improvement in ambulatory function with an anti-inflammatory disease-modifying agent highlights the need for therapies that enhance endogenous repair or improve functionality through non–anti-inflammatory mechanisms.

Most SPMS trials with disease-modifying agents have focused on slowing disability progression as determined by the EDSS [24]–[26] despite the known poor responsiveness of the EDSS to disease progression in the higher EDSS range that is characteristic of SPMS [11], [27]. The IMPACT study was different because it used the MSFC as the primary outcome and showed statistically significant treatment effects of IM IFNβ-1a observed as early as month 3 after initiation of therapy [14].

There have been previous publications regarding improvement of ambulation in MS with natalizumab. A post hoc responder analysis of all RRMS subjects enrolled in AFFIRM showed that 45% of subjects with baseline T25FW >5 seconds randomized to natalizumab improved their T25FW performance in at least 7 of 10 assessments over 2 years compared with 28% in the placebo arm, an absolute difference of 17% (*P<*0.001) [28]. Another post hoc analysis of AFFIRM revealed that natalizumab treatment increased the cumulative probability of 3-month confirmed EDSS improvement over 2 years by 69% compared with placebo (hazard ratio = 1.69; 95% confidence interval, 1.16–2.45; *P = *0.006) [7]. Improvement with natalizumab was recently shown on abnormal electrophysiological parameters including visual and somatosensory evoked potentials [29].

Although natalizumab has not been thoroughly studied in the context of SPMS, the current analyses suggest it may have the potential to improve walking ability in SPMS. This requires confirmation in a prospective clinical trial. One way natalizumab could do this is by reducing the intrathecal inflammation that has been implicated in the pathogenesis of SPMS [30]–[33], which in turn could enable endogenous repair to take place. A recent autopsy study showed a close association between neuroinflammation and neurodegeneration in all MS lesions and disease stages [34]. The observation that natalizumab normalizes cerebrospinal fluid levels of the ectopic lymphoid organ chemokine CXCL13 in RRMS provides one potential mechanism natalizumab could be beneficial in SPMS; cerebrospinal fluid CXCL13 is elevated in SPMS [35]. The mechanism of action that leads to walking improvement with natalizumab is likely due to its anti-inflammatory effect that enables endogenous repair, while walking improvement with PR fampridine is quite different, involving modulation of presynaptic potassium channels in demyelinating CNS axons [36].

There are several limitations to our analysis including that it is post hoc, that there is variability of the frequency and length of administration of the T25FW in the various trials, and that the data suggesting efficacy in SPMS come from early-phase studies with small sample sizes and short durations. Furthermore, one of the studies included, DELIVER, did not have a placebo group, and its pooled natalizumab groups were treated with the same dose given by different routes of administration. However, these preliminary clinical observations raise the intriguing possibility that natalizumab may be efficacious not only in disabled RRMS but also in SPMS patients. A large, registrational, prospective, placebo-controlled SPMS clinical trial (ASCEND, NCT# 01416181) is currently underway to investigate this possibility.

## Supporting Information

Appendix S1 **Independent ethics committees/institutional review boards for participating study centers in the DELIVER study.**
(DOCX)
