# Supplementary material for: Impact of Natalizumab on Ambulatory Improvement in Secondary Progressive and Disabled Relapsing-Remitting Multiple Sclerosis
Source: PLoS One. 2013 Jan 4;8(1):e53297. doi: 10.1371/journal.pone.0053297 (PMC3537666; doi:10.1371/journal.pone.0053297)
Supplement: Appendix S1 — Independent ethics committees/institutional review boards for participating study centers in the DELIVER study. (DOCX) [file pone.0053297.s001.docx]

**Appendix S1.** Independent ethics committees/institutional review boards for participating study centers in the DELIVER study.

Alta Bates Institutional Review Board

2450 Ashby Avenue

Berkeley, CA 94705

Aspire Institutional Review Board–San Diego

9320 Fuerte Drive, Suite 105

La Mesa, CA 91941

Aurora Health Care Research Subject Protection Program

945 North 12th Street

PO Box 342, W310

Milwaukee, WI 53201

Health Sciences Institutional Review Board

University at Buffalo, The State University of New York

150 Parker Hall

3435 Main Street

Buffalo, NY 14214-8004

Mayo Clinic Institutional Review Board

200 First Street SW

201 Building, Room 4-60

Rochester, MN 55905

Mount Sinai School of Medicine Institutional Review Board

One Gustave L. Levy Place

ICAHN Building, Box 1075

New York, NY 10029

Peoria Institutional Review Board

1 Illini Drive

PO Box 1649

Peoria, IL 61656

Winthrop University Hospital Institutional Review Board

222 Station Plaza North, Suite 521

Mineola, NY 11501
